# Prevalence and Prognostic Role of *BRCA1/2* Variants in Unselected Chinese Breast Cancer Patients

**DOI:** 10.1371/journal.pone.0156789

**Published:** 2016-06-03

**Authors:** Xiaorong Zhong, Zhengwei Dong, Hua Dong, Jiayuan Li, Zuxiang Peng, Ling Deng, Xuehua Zhu, Yun Sun, Xuesong Lu, Fuxiao Shen, Xinying Su, Liying Zhang, Yi Gu, Hong Zheng

**Affiliations:** 1 Laboratory of Molecular Diagnosis of Cancer, State Key Laboratory of Biotherapy, National Collaborative Innovation Center for Biotherapy, West China Hospital, Sichuan University, Chengdu Sichuan, P. R. China; 2 Asia and Emerging Markets iMed, AstraZeneca, Shanghai, P. R. China; 3 Department of Epidemiology and Bio-Statistics, West China School of Public Health, Sichuan University, Chengdu, Sichuan, P.R. China; 4 Department of Thyroid and Breast Surgery, West China Hospital, Sichuan University, Chengdu Sichuan, P. R. China; 5 Department of Pathology, Memorial Sloan Kettering Cancer Center, New York, NY, United States of America; 6 Cancer center, West China Hospital, Sichuan University, Chengdu, Sichuan, P. R. China; CNR, ITALY

## Abstract

**Background:**

The prevalence of *BRCA1/2* variants in Chinese breast cancer patients varies among studies. Germline or somatic *BRCA1/2* mutations are associated with sensitivity to poly(ADP-ribose) polymerase-1 inhibitors and DNA-damaging agents. We aimed to investigate the distribution of both somatic and germline *BRCA1/2* variants in unselected Chinese breast cancer patients, and explore their roles in tumor phenotype and disease prognosis.

**Methods:**

507 breast cancer patients, unselected for family history of breast cancer or age at diagnosis, were prospectively enrolled from West China Hospital between Feb. 2008 and Feb. 2014. *BRCA1/2* variants in the exons/flanking regions were detected in fresh-frozen tumors using next-generation sequencing and confirmed by independent methods. Germline/somatic status was validated by Sanger sequencing in paired blood/normal tissue.

**Results:**

*BRCA1/2* pathogenic or likely pathogenic (P/LP) variants were detected in 50 patients (9.9%), including 40 germline carriers (18 in *BRCA1*, 22 in *BRCA2*), 9 patients with somatic variants (3 in *BRCA1*, 6 in *BRCA2*), and 1 patient with concurrent germline/somatic variants in *BRCA2*. The triple-negative (21.4%) and Luminal B (9.7%) subtypes had higher rates of *BRCA1/2* variants. In patients with disease stage 0~II, presence of a germline or somatic *BRCA1* P/LP variant increased the risk of relapse as compared to non-carriers [univariate hazard ratio (HR): 3.70, *P* = 0.04]. Germline *BRCA1* P/LP variants, which were associated with aggressive tumor phenotypes, predicted worse disease-free survival in the subgroup of stage 0~II (HR: 4.52, *P* = 0.02) and N0 (HR: 5.4, *P* = 0.04) compared to non-carriers.

**Conclusion:**

A high frequency of germline and somatic *BRCA1/2* P/LP variants was detected in unselected Chinese breast cancer patients. Luminal B subtype should be considered as a high-risk population of *BRCA1/2* mutation, in addition to triple-negative breast cancer. *BRCA1* status was associated with aggressive tumor phenotype and worse disease progression in early stage breast cancer patients.

## Introduction

Germline mutations in breast cancer susceptibility genes *BRCA1* and *BRCA2* confer a high risk of breast and ovarian cancer. At the age of 70 years old, the mean cumulative breast cancer risks for *BRCA* mutation carriers are 47%-66% (*BRCA1*) and 40%-57% (*BRCA2*) in western countries [1]. Around 25% of familial breast cancer (FBC) may be attributed to inherited mutations in *BRCA1* and *BRCA2* [2]. The prevalence of germline *BRCA1/2* mutations in Chinese breast cancer patients varies among the previous studies (1.8%~18.2%)[3–5], largely due to differences in patient selection criteria, sensitivity and specificity of detection assays, and mutation calling methods. Therefore, the prevalence of *BRCA1/2* mutation in unselected Chinese breast cancer patients has not been fully explored.

*BRCA1* and *BRCA2* play important roles in maintaining genome integrity by acting at different stages in the DNA damage response and DNA repair processes [6]. Recent clinical evidence showed that cancer patients with *BRCA1/2* mutations (germline or somatic) were particularly sensitive to poly(ADP-ribose) polymerase inhibitors (PARPi)[7–8] and DNA damaging chemotherapy (e.g., platinum [9]). In a phase 2 trial, Ledermann and colleagues [10] reported that olaparib maintenance monotherapy significantly prolonged progression-free survival (PFS) in patients with platinum-sensitive recurrent serous ovarian cancer. Further subgroup analysis suggested that the patients with a germline or somatic *BRCA1/2* mutation had the most profound benefit from olaparib, which significantly extended their median PFS by 6.9 months, as compared to 1.9 months increase in wild-type *BRCA* patients. Based on these findings, the European Medicines Agency (EMA) included both germline and somatic *BRCA1/2* mutation patients with ovarian cancer for olaparib maintenance monotherapy. Other additional study of sporadic cancers with a somatically mutated *BRCA* gene showed the similar phenotype (*BRCA*ness) to germline *BRCA* tumors, in terms of their treatment susceptibility to DNA-damaging agents [11]. Thus, it is equally important to detect germline and somatic *BRCA* mutations to identify the population that would most benefit from the DNA-damaging therapy.

In breast cancer patients, the relationship between *BRCA* status and prognosis was complex [12–14]. Triple-negative breast cancer patients carrying founder *BRCA1* mutations were likely to had a decreased risk of distant recurrence and breast cancer-specific mortality compared to *BRCA1* non-carriers [15]. On the other hand, a recent meta-analysis from 13 studies of 10,016 women with breast cancer [16] concluded that *BRCA1* mutation carriers had a worse overall survival but similar progression-free survival, compared to non-carriers; while *BRCA2* mutation was not associated with breast cancer prognosis.

In this single-center observational study, we aimed to investigate the prevalence of both somatic and germline *BRCA1/2* variants in unselected Chinese breast cancer patients, and explore their role in tumor phenotype and disease prognosis. We evaluated the frequency of likely pathogenic or pathogenic *BRCA1/2* variants in tumor tissues from 507 Chinese breast cancer patients using next generation sequencing (NGS). All these variants were subsequently confirmed in the tumors and the paired blood/normal tissues by Sanger sequencing or other methods. We further assessed the clinical characteristics of *BRCA1* and *BRCA2* carriers by germline/somatic status. The disease-free survival (DFS) and overall survival (OS) patterns for genetically different patients were also explored.

## Materials and Methods

### Study patients and samples

Patients pathologically diagnosed with breast cancer were prospectively registered in the Breast Cancer Information Management System (BCIMS) at West China Hospital, Sichuan University since 2008. Medical records, diagnostic pathology reports, treatments records were reviewed and collected by oncologists. All patients were followed by outpatient visit or telephone at 3 to 4-month intervals within 3 years after diagnosis, 6-month intervals within 4–5 years, and then annually. Between Feb. 2008 and Feb. 2014, there were a total of 5103 patients registered, about 600 to 900 new cases per year. The rate of loss to follow-up was around 10% and less than 5% in patients diagnosed between 2008~2009 and 2010~2014, respectively.

This study aimed to fully understand the distribution of *BRCA1/2* gene variants, both somatic and germline, in unselected breast cancer patients, as well as their potential impact on disease prognosis. Among the registered patients, 4791 cases who had undergone surgery in the Department of Thyroid and Breast Surgery between Feb. 2008 and Feb. 2014, regardless of their family history of breast cancer or age at diagnosis, were recruited. However, 3779 patients who were unable to provide sufficient amount of frozen tumor tissue or didn’t have complete clinical information, were excluded from this study. Another 422 patients without the matched frozen distal adjacent normal tissue or peripheral blood were also excluded. The neoplastic cellularity of tumor tissue section from 83 patients didn’t meet the quality control criteria of 50% or greater tumor content. Finally, 507 patients were eligible for *BRCA* test and clinical characteristics analysis. Survival analysis was performed on 426 patients of stage 0~III, excluding 5 patients with stage IV, 4 patients with unspecified stage, and 72 patients with the variants of uncertain significance (VUS). This study was approved by the Clinical Test and Biomedical Ethics Committee of West China Hospital, Sichuan University. Written informed consent was provided by all the patients.

### Targeted DNA sequencing and variant interpretation

Details of the comprehensive NGS workflow for testing and analyzing tumor *BRCA1/2* variants were described in the S1 Method and our previous work [17]. Briefly, tumor DNA samples were screened for variants in all coding exons and the splice boundaries (-20/+10 bp) of *BRCA1* and *BRCA2* genes using NGS on a MiSeq system (Illumina, USA). All variant candidates were successfully validated in tumors using Sanger sequencing, or the MassARRAY system (Sequenom, USA), or long-range PCR. Somatic or germline status was confirmed by testing the paired blood or normal tissue sample using Sanger sequencing.

Germline variants were interpreted according to the American College of Medical Genetics and Genomics (ACMG) [18]. Briefly, variants that produce premature termination codons which are associated with non-functional or truncated proteins were classified as pathogenic (P) variants: such as nonsense mutations, frameshift mutations, splice site mutations and exonic deletions. Some missense mutations were considered as likely pathogenic (LP) variants based on available evidence indicating a strong likelihood of their association with disease. Variants with undetermined clinical significance were classified as VUS: such as novel point mutations, certain missense variants, and variants located in intronic regions. Similarly, inactivating somatic variants were considered as pathogenic variants: such as nonsense mutations and frameshift mutations, while somatic variants with uncertain clinical significance were considered as VUS: such as missense variants.

### LOH analysis

The heterozygosity state of the *BRCA1/2* pathogenic/likely pathogenic (P/LP) variants was determined by Sanger sequencing using the genomic DNA from tumors and paired blood/normal tissue. Loss of heterozygosity (LOH) in tumor was defined as the presence of heterozygosity in the blood/normal tissue, but not in the tumor. LOH analysis was performed in 40 patients carrying germline *BRCA1/2* P/LP variants.

### Clinical and survival data

Clinical and pathological characteristics of 507 patients were extracted from the BCIMS (S1 Table). Immunohistochemistry (IHC) scoring for estrogen receptor (ER), progesterone receptor (PR) was performed according to the Guidelines for Testing of ER and PR in Breast Cancer [19]; IHC and fluorescence in situ hybridization scoring for human epidermal growth factor receptor-2 (HER2) was conducted following the Guidelines for HER2 Detection in Breast Cancer[20]. Standard therapy was defined as administration of comprehensive therapy according to National Comprehensive Cancer Network Guidelines (NCCN, http://www.nccn.org) and St. Gallen International Expert Consensus[21]. In this study, 426 patients of stage 0~III were followed for up to seven years (median, 39.3 months). The last follow-up date was April 1, 2015. DFS was defined as the interval between surgery date and first relapse of cancer, breast cancer-related death, or last follow-up. OS was defined as the interval between surgery date and death as a result of disease, or last follow-up.

### Statistical analysis

Comparison of clinical characteristics between patients with and without *BRCA1/2* variants (non-carriers) was performed using two-tailed t-tests, Pearson Chi-Square tests, or Fisher’s exact tests as appropriate (SPSS version 20; SPSS, Chicago, IL). Missing data was not included in the analysis. Survival analyses were performed using univariate and multivariate Cox proportional hazards regression models (SPSS version 20). The log-rank test and Kaplan-Meier plots were used to visualize survival characteristics (STATA version 12; StataCorp, College Station, TX). A two-sided test *P* values < 0.05 were considered as statistically significant.

## Results

### Prevalence and characteristics of *BRCA1/2* variants

The prevalence, classification and germline/somatic status of the *BRCA1/2* variants identified in this cohort were summarized in Table 1. Among the 507 unselected breast cancer patients, 40 (7.9%) had a germline *BRCA1/2* P/LP variant, and 9 (1.8%) had a somatic *BRCA1/2* pathogenic variant. 1 (1.8%) patient had two pathogenic *BRCA2* variants: 1 germline and 1 somatic. Thus, the total percentage of patients with germline or somatic *BRCA1/2* P/LP variants was 9.9%.

**Table 1:** Distribution of germline or somatic *BRCA1/2* variants in 507 breast cancer patients.

Gene	Pathogenic/Likely pathogenic (P/LP) variant		Variant of uncertain significance (VUS) ^b^	
	Patient No.	Percentage ^a^	Patient No.	Percentage ^a^
**Germline**				
***BRCA1***	18	3.6%	16	3.2%
***BRCA2***	22	4.3%	43	8.5%
***BRCA1* and *BRCA2* concurrent**	0	0.0%	3	0.6%
**Subtotal**	40	7.9%	62	12.2%
**Somatic**				
***BRCA1***	3	0.6%	3	0.6%
***BRCA2***	6	1.2%	5	1.0%
**Subtotal**	9	1.8%	8	1.6%
**Germline and somatic concurrent**				
***BRCA1***	0	0.0%	0	0.0%
***BRCA2***	1	0.2%	0	0.0%
***BRCA1* and *BRCA2* concurrent**	0	0.0%	1	0.2%
**Subtotal**	1	0.2%	1	0.2%
**Total**	50	9.9%	71	14.0%

^a^ The percentage of carriers out of 507 breast cancer patients.

^b^ One *BRCA2* VUS was not listed in this table due to its unknown germline/somatic status.

The variant data has been submitted to NCBI ClinVar database (http://www.ncbi.nlm.nih.gov/clinvar/, Submission ID: SUB1362368, Submission name: Chinese507BC_BRCA).

#### Germline BRCA1/2 variants

Germline *BRCA1* P/LP variants were simultaneously detected in tumors and paired blood/normal tissue from 18 (3.6%) patients, and the germline *BRCA2* P/LP variants were found in 22 (4.3%) patients. Among them, LOH in tumor was identified in 11 (61.1%) *BRCA1* carriers and 13 (59.1%) *BRCA2* carriers, respectively. There are 39 unique germline P/LP variants in this cohort. Half of them (20/39) are novel, including 14 frameshift insertion/deletion, 4 nonsense mutation, 1 splice site mutation, and 1 missense mutation (S2 Table). Also, we found a *BRCA1* founder mutation (c.981_982del), which was previously reported in Southern Chinese breast cancer patients [22]. In addition, 12.2% of the patients in this cohort had germline VUS in *BRCA1/2* genes (Table 1 and S2 Table).

Next, we compared the clinical characteristics between 40 germline *BRCA* variants carriers and 385 non-carriers (Table 2). The *BRCA1/2* carriers (mean±standard deviation: 46.6±8.5 years), especially *BRCA2* (46.2±8.0 years), had an earlier onset age than non-carriers (50.7±10.3 years), which supports *BRCA* genes as potential cancer risk factors. The majority of *BRCA1* (18/18) and *BRCA2* (19/22) carriers were identified from the patients without family history of breast or ovarian cancer. The remaining 3 *BRCA2* carriers were found among the 16 FBC patients.

**Table 2:** A comparison between clinical characteristics of germline *BRCA1/2* variant carriers and non-carriers.

Characteristics	Non-carriers No. (n = 385)	Germline *BRCA1/2* P/LP variants carriers No. (n = 40)						Germline *BRCA1/2* VUS carriers No. (n = 62)
		*BRCA1/2* (n = 40)	*P* ^a^	*BRCA1* (n = 18)	*P* ^a^	*BRCA2* (n = 22)	*P* ^a^	
**Age at diagnosis (y, mean±SD)**	50.7±10.3	46.6±8.5	**0.01**	46.9±9.4	0.13	46.2±8.0	**0.05**	48.5±9.2
**Family history of breast cancer**								
**Yes**	12	3	0.16	0	1.00	3	**0.04**	1
**No**	373	37		18		19		61
**Menopause at diagnosis**								
**Postmenopause**	176	13	0.10	6	0.29	7	0.19	23
**Premenopause**	206	27		12		15		39
**Molecular subtype**								
**Luminal A**	49	1	**< 0.001**	0	**< 0.001**	1	0.41	10
**Luminal B**	218	21		4		17		35
**HER2+**	57	1		0		1		8
**TN**	48	14		11		3		6
**ER-PR+**	13	3		3		0		3
**ER**								
**Positive (>1%)**	274	22	**0.03**	4	**< 0.001**	18	0.28	47
**Negative**	111	18		14		4		15
**PR**								
**Positive (>1%)**	266	22	0.07	6	**0.002**	16	0.72	43
**Negative**	119	18		12		6		19
**Ki67**								
**<14%**	68	1	**0.01**	0	**0.05**	1	0.15	12
**≥14%**	315	39		18		21		50
**HER2**								
**Positive**	130	2	**< 0.001**	0	**0.003**	2	**0.02**	18
**Negative**	255	38		18		20		44
**Histology**								
**DCIS**	3	0	**0.05**	0	0.34	0	0.28	2
**Invasive ductal carcinoma**	341	40		18		22		53
**Other Invasive carcinomas**	41	0		0		0		7
**Histological grade**								
**I/II**	126	8	0.06	1	**0.01**	7	0.73	25
**III**	230	31		16		15		33
**T**								
**Tis/T1**	128	11	0.76	4	0.70	7	1.00	20
**T2**	227	26		13		13		36
**T3**	17	1		1		0		3
**T4**	11	0		0		0		3
**No. of lymph node involvement**								
**0**	166	22	0.28	11	0.32	11	0.71	33
**1~3**	133	10		4		6		22
**≥4**	85	7		3		4		7
**M**								
**M0**	381	38	0.07	16	**0.02**	22	1.00	62
**M1**	3	2		2		0		0
**Clinical stage**								
**0/I**	60	7	0.13	2	**0.04**	5	0.62	12
**II**	227	22		11		11		40
**III**	93	7		3		4		10
**VI**	3	2		2		0		0
**Standard therapy**								
**Yes**	15	2	0.67	0	1.00	2	0.23	4
**No**	370	38		18		20		58

^a^ *P*-value calculated comparing with non-carriers by two-tailed t-tests, Pearson Chi-Square tests, or Fisher's Exact tests as appropriate. Unknown data were not included in the analysis.

Among different molecular subtypes, germline *BRCA1/2* P/LP variants were preferentially detected in the patients with triple-negative (14/70; 20.0%) and Luminal B (21/290; 7.2%) breast cancer, as compared to the patients of Luminal A (1/60; 1.7%) and HER2 positive (1/67; 1.5%). Interestingly, we found that in TNBC subtype, 11 out of the 14 patients with *BRCA1/2* P/LP variants were *BRCA1* carriers. Vice Versa in Lumina B subtype, 17 out of the 21 patients with *BRCA1/2* P/LP variants were *BRCA2* carriers. *BRCA1* carriers were more likely associated with ER negative (77.7% vs. 28.8%, *P*<0.001), PR negative (66.7% vs. 30.9%, *P* = 0.002), HER2 negative (100.0% vs. 66.2%, *P* = 0.003), and Ki67 overexpression (100.0% vs. 82.2%, *P* = 0.05) as compared to non-carriers.

Furthermore, we found that *BRCA1* carriers had a higher chance of the histological grade III tumors than non-carriers. Metastasis at diagnosis was found in 2 patients of the 16 *BRCA1* carriers, again significantly higher than that of non-carriers (3/384). In addition, all the germline *BRCA1/2* P/LP variants were found in the patients with invasive ductal carcinoma. No significant differences were found in tumor size, lymph node involvement, or menopause at diagnosis.

#### Somatic BRCA1/2 variants

9 somatic pathogenic variants were detected in tumors, which didn’t appear in paired blood/normal tissues: 3 (0.6%) in *BRCA1* gene and 6 (1.2%) in *BRCA2* gene. Among those 9 unique variants, 4 frameshift insertion/deletions are novel (S2 Table). Only 1.6% of the 507 patients had somatic VUS in *BRCA1/2* genes (Table 1 and S2 Table).

In addition, patients with somatic *BRCA1/2* P variants were all HER-2 negative (*P* = 0.03; Table 3), but had no significant differences in other clinical characteristics, compared to non-carriers.

**Table 3:** A comparison between clinical characteristics of patients with somatic *BRCA1/2* variants and non-carriers.

Characteristics	Non-carriers No. (n = 385)	No. of patients with somatic *BRCA1/2* P variants (n = 9)						No. of patients with Somatic *BRCA1/2* VUS (n = 8)
		*BRCA1/2* (n = 9)	*P* ^a^	*BRCA1* (n = 3)	*P* ^a^	*BRCA2* (n = 6)	*P* ^a^	
**Age at diagnosis (y, mean±SD)**	50.7±10.3	49.7±10.4	0.77	42.0±12.1	0.15	53.5±7.9	0.51	51.5±6.0
**Family history of breast cancer**								
**Yes**	12	0	1.00	0	1.00	0	1.00	0
**No**	373	9		3		6		8
**Menopause at diagnosis**								
**Postmenopause**	176	5	0.74	1	1.00	4	0.42	4
**Premenopause**	206	4		2		2		4
**Molecular subtype**								
**Luminal A**	49	0	0.29	0	0.11	0	0.63	0
**Luminal B**	218	7		1		6		7
**HER2+**	57	0		0		0		0
**TN**	48	1		1		0		1
**ER-PR+**	13	1		1		0		0
**ER**								
**Positive(>1%)**	274	7	1.00	1	0.20	6	0.19	7
**Negative**	111	2		2		0		1
**PR**								
**Positive(>1%)**	266	7	0.73	2	1.00	5	0.67	5
**Negative**	119	2		1		1		3
**Ki67**								
**<14%**	68	2	0.67	1	0.45	1	1.00	0
**≥14%**	315	7		2		5		8
**HER2**								
**Positive**	130	0	**0.03**	0	0.55	0	0.18	2
**Negative**	255	9		3		6		6
**Histology**								
**DCIS**	3	0	1.00	0	1.00	0	0.52	0
**Invasive ductal carcinoma**	341	8		3		5		8
**Other Invasive carcinomas**	41	1		0		1		0
**Histological grade**								
**I/II**	126	2	0.50	1	1.00	1	0.67	2
**III**	230	7		2		5		6
**T**								
**Tis/T1**	128	0	0.10	0	0.65	0	0.30	1
**T2**	227	9		3		6		7
**T3**	17	0		0		0		0
**T4**	11	0		0		0		0
**No. of lymph node involvement**								
**0**	166	4	0.56	2	0.45	2	0.77	2
**1~3**	133	2		0		2		2
**≥4**	85	3		1		2		4
**M**								
**M0**	381	9	1.00	3	1.00	6	1.00	8
**M1**	3	0		0		0		0
**Clinical stage**								
**0/I**	60	0	0.48	0	1.00	0	0.74	1
**II**	227	6		2		4		3
**III**	93	3		1		2		4
**VI**	3	0		0		0		0
**Standard therapy**								
**Yes**	370	38	0.67	18	1.00	20	0.23	58
**No**	15	2		0		2		4

^a^ *P*-value calculated comparing with non-carriers by two-tailed t-tests and Fisher's Exact tests as appropriate. Unknown data were not included in the analysis.

### Association of *BRCA1/2* P/LP variants with survival

Survival analysis was conducted among 426 patients of stage 0~III, including 19 *BRCA1* carriers (16 germline, 3 somatic), 27 *BRCA2* carriers (20 germline, 6 somatic, 1 concurrent) and 380 non-carriers. The standard comprehensive therapy patterns were similar between *BRCA1/2* carriers and non-carriers (Tables 2 and 3). In *BRCA1* group, relapse occurred in 3 germline carriers, and no death were reported; while no relapse or death occurred in the *BRCA2* group during the follow-up period. In the non-carriers group, 35 relapses and 20 deaths occurred.

#### Disease-free survival of stage 0~III patients

**Fig 1:**
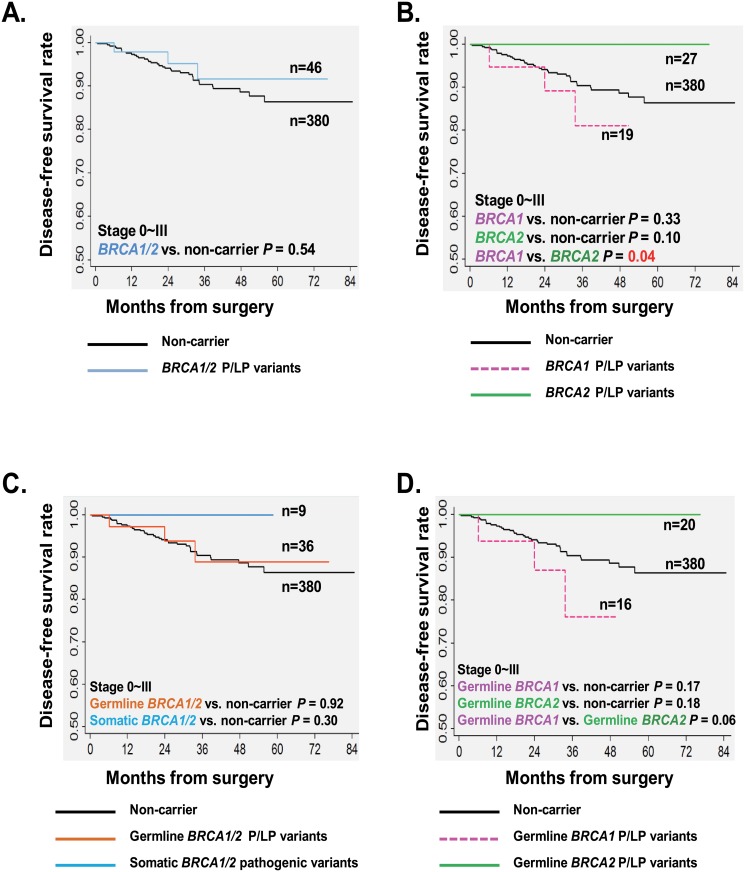
Impact of *BRCA1/2* P/LP variants on the DFS of BC patients with stage 0~III. Kaplan-Meier survival analysis; *P* values calculated using a log-rank analysis. Estimated DFS of breast cancer patients with stage 0~III by *BRCA* status: (A) *BRCA1/2* P/LP variants (germline or somatic); (B) *BRCA1* and *BRCA2* P/LP variants (germline or somatic); (C) Germline and somatic *BRCA1/2* P/LP variants; (D) Germline *BRCA1* and germline *BRCA2* P/LP variants.

As shown in Fig 1A, the stage 0~III patients with a germline or somatic *BRCA1/2* P/LP variant had a similar estimated 3-year DFS rate with non-carriers (91.7%±4.7% vs. 90.4%±1.7%; log-rank *P* = 0.54). Interestingly, *BRCA1* carriers (germline or somatic) had an inferior 3-year DFS rate than *BRCA2* carriers (81.1%±10.2% vs. 100%; log-rank *P* = 0.04; Fig 1B). In univariate Cox proportional hazards regression models, the presence of a germline or somatic *BRCA1/2* variant was not associated with DFS [hazard ratio (HR) for *BRCA1/2* = 0.69, *P* = 0.54; HR for *BRCA1* = 1.78, *P* = 0.34; HR for *BRCA2* = 0, *P* = 0.98; Table 4]. Instead, postmenopause (HR = 2.22, *P* = 0.02), and higher tumor grade (HR for grade III = 3.54, *P* = 0.008) predicted high risks of relapse; while early disease stages predicted lower risks of relapse (HR for stage 0/I = 0.15, *P* = 0.01; HR for stage II = 0.39, *P* = 0.004; Table 4).

**Table 4:** Impact of *BRCA1/2* P/LP variants and other variables on the DFS and OS of breast cancer patients (Stage 0~III).

Variables	DFS			OS		
	#Cases (events)	HR (95% CI) ^a^	*P*	#Cases (deaths)	HR (95% CI) ^a^	*P*
**Germline and somatic combined variants**						
**Non-carrier**	380 (35)	1		380 (20)	1	
***BRCA1/2***	46 (3)	0.69 (0.21–2.25)	0.54	46 (0)	0	0.30
***BRCA1***	19 (3)	1.78 (0.55–5.81)	0.34	19 (0)	0	0.51
***BRCA2***	27 (0)	0	0.98	27 (0)	0	0.42
**Germline variants**						
**Non-carrier**	380 (35)	1		380 (20)	1	
***BRCA1/2***	36 (3)	0.94 (0.29–3.06)	0.92	36 (0)	0	0.37
***BRCA1***	16 (3)	2.22 (0.68–7.26)	0.19	16 (0)	0	0.55
***BRCA2***	20 (0)	0	0.97	20 (0)	0	0.50
**Somatic variants**						
**Non-carrier**	380 (35)	1		380 (20)	1	
***BRCA1/2***	9 (0)	0	0.49	9 (0)	0	0.60
**Age at diagnosis**	426 (38)	1.01 (0.98–1.05)	0.38	426 (20)	1.03 (0.99–1.08)	0.11
**Menopause at diagnosis**						
**Postmenopause**	188 (24)	**2.22 (1.15–4.29)**	**0.02**	188 (15)	**3.81 (1.38–10.49)**	**0.01**
**Premenopause**	235 (14)	1		235 (5)	1	
**Molecular subtype**						
**Luminal A**	49 (2)	0.28 (0.06–1.3)	0.10	49 (1)	0.26 (0.03–2.34)	0.23
**Luminal B**	241 (16)	0.48 (0.21–1.12)	0.09	241 (8)	0.46 (0.14–1.53)	0.20
**HER2+**	59 (8)	1.00 (0.37–2.66)	1.00	59 (4)	0.95 (0.24–3.83)	0.95
**TN**	60 (8)	1		60 (4)	1	
**ER-PR+**	17 (4)	1.9 (0.57–6.31)	0.30	17 (3)	2.62 (0.58–11.75)	0.21
**Histological grade**						
**I/II**	133 (5)	1		133 (3)	1	
**III**	264 (33)	**3.54 (1.38–9.07)**	**0.008**	264 (17)	2.92 (0.86–9.99)	0.09
**Clinical stage**						
**0/I**	67 (2)	**0.15 (0.04–0.67)**	**0.01**	67 (0)	0	0.96
**II**	256 (18)	**0.39 (0.2–0.74)**	**0.004**	256 (8)	**0.26 (0.11–0.64)**	**0.003**
**III**	103 (18)	1		103 (12)	1	
**Standard therapy**						
**No**	16 (3)	1		16 (2)	1	
**Yes**	410 (35)	0.32 (0.1–1.03)	0.06	410 (18)	**0.22 (0.05–0.97)**	**0.05**

^a^ Univariate Cox proportional hazards regression models

Next, we investigated the impact of the germline and somatic variants separately. Interestingly, the germline *BRCA1* carriers showed a lower 3-year DFS rate than the germline *BRCA2* carriers (76.2%±12.6% vs. 100%; log-rank *P* = 0.06; Fig 1D). However, there were no significant difference among 3-year DFS rates for germline *BRCA1/2* group (88.8%±6.3%), somatic *BRCA1/2* group (100%) and non-carriers (90.4%±1.7%) (Fig 1C). No significant association was found between the risk of relapse with germline *BRCA1/2* variants or somatic ones in this general population of stage 0~III (Table 4).

#### Disease-free survival of subgroups by clinical stage

**Fig 2:**
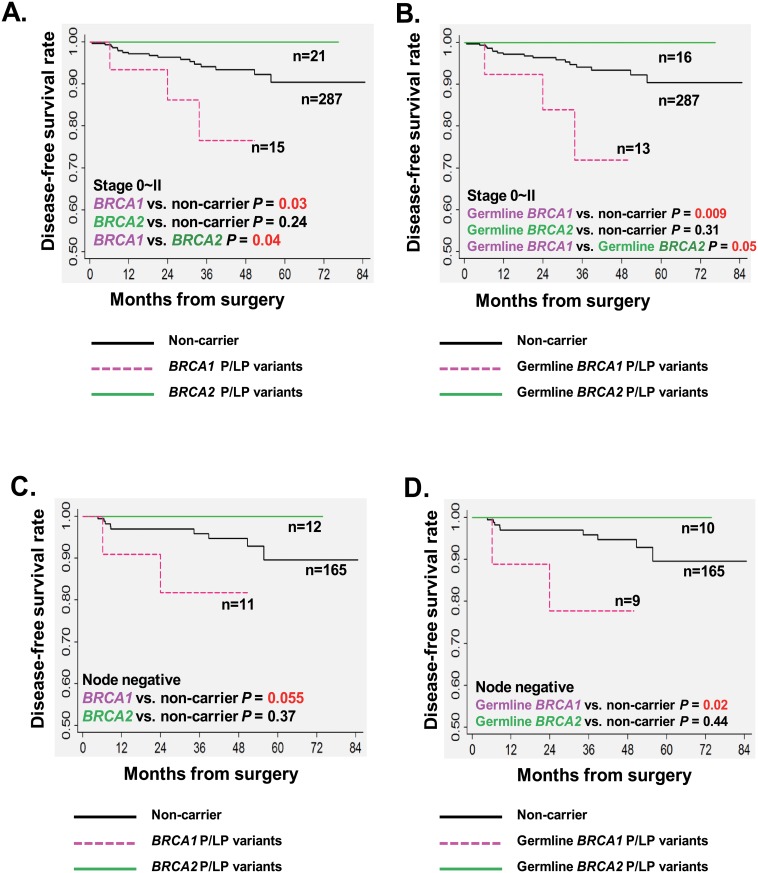
Impact of *BRCA1/2* P/LP variants on the DFS of subgroups with early stage. Kaplan-Meier survival analysis; *P* values calculated using a log-rank analysis. Estimated DFS of breast cancer patients with stage 0~II by *BRCA* status: (A) *BRCA1* and *BRCA2 P/LP* variants (germline or somatic); (B) Germline *BRCA1* and *BRCA2* P/LP variants. Estimated DFS of breast cancer patients with node negative disease by *BRCA* status: (C) *BRCA1* and *BRCA2* P/LP variants (germline or somatic); (D) Germline *BRCA1* and *BRCA2* P/LP variants.

We further conducted stratified survival analysis by disease stages. In the subgroup with a relatively earlier stage (0~II), we observed that 3-year DFS rate of the 15 *BRCA1* carriers (germline or somatic) was significantly worse than 287 non-carriers (76.6%±12.1% vs. 94.1%±1.6%; log-rank *P* = 0.03), and also worse than 21 *BRCA2* carriers (germline or somatic) (76.6%±12.1% vs. 100%; log-rank *P* = 0.04; Fig 2A). Moreover, the univariate analysis demonstrate that *BRCA1* variants significantly increased the relapse risk by 3.7 fold as compared to non-carriers [HR = 3.70; 95% confidence interval (CI) = 1.08–12.76; *P* = 0.04; Table 5]. However, the statistical significance didn’t maintain when HR was adjusted by classic prognostic factors, including age at diagnosis, molecular subtype, tumor grade and administration of standard therapy (HR = 2.06;95% CI = 0.48–8.8;P = 0.33; Table 5).

**Table 5:** Impact of *BRCA1/2* P/LP variants on the DFS of breast cancer patients from subgroups of stage 0~II and III.

P/LP variants	#Cases (events)	HR (95% CI) ^a^	*P* ^a^	Adjusted HR (95% CI) ^b^	*P* ^b^
**Stage 0~II**					
**Germline and somatic variants combined**					
**Non-carrier**	287 (17)	1		1	
***BRCA1***	15 (3)	**3.70 (1.08–12.76)**	**0.04**	2.06 (0.48–8.8)	0.33
***BRCA2***	21 (0)	0	0.98	0	0.99
**Germline variants**					
**Non-carrier**	287 (17)	1		1	
***BRCA1***	13 (3)	**4.52 (1.31–15.61)**	**0.02**	2.72 (0.65–11.44)	0.17
***BRCA2***	16 (0)	0	0.98	0	0.99
**Somatic variants**					
**Non-carrier**	287 (17)	1		1	
***BRCA1/2***	6 (0)	0	0.66	0	1.00
**Stage III**					
**Germline and somatic variants combined**					
**Non-carrier**	93 (18)	1		1	
***BRCA1***	4 (0)	0	0.55	0	0.99
***BRCA2***	6 (0)	0	0.51	0	0.99

^a^ Univariate Cox proportional hazards regression models

^b^ Multivariate Cox proportional hazards regression models. HR was adjusted by age at diagnosis, molecular subtype (Luminal A, Luminal B, TNBC, HER2+ and ER-PR+), tumor grade (grade I/II and III) and administration of standard therapy.

In the subgroup analysis for stage 0~II, patients with a germline *BRCA1* variant had a significantly worse 3-year DFS rate of 71.9%±14.3%, compared with 94.1%±1.6% for non-carriers (*P* = 0.009), and 100% for ones with a germline *BRCA2* variant (*P* = 0.05) (Fig 2B). The presence of a germline *BRCA1* variant particularly predicted an increased risk of relapse as compared to non-carriers (unadjusted HR = 4.52;95% CI = 1.31–15.61;P = 0.02; Table 5). However, no events occurred in patients with somatic *BRCA1/2* variants, and no impact on the relapse risk was observed (Table 5).

On the other hand, we didn’t find any prediction role of *BRCA1* or *BRCA2* variants (germline or somatic) in relapse risk for the subgroup of stage III (Table 5).

#### Disease-free survival of subgroups by N stage

In stratified analysis by lymph node involvement, we also found an inferior DFS in 11 *BRCA1* carriers (germline or somatic) than 165 non-carriers with a marginal significance in the patients without lymph node involvement (3-year DFS rate: 81.8%±11.6% vs. 95.9%±1.7%; log-rank *P* = 0.055; Fig 2C). A trend of an increased risk of relapse was found in *BRCA1* carriers by univariate analysis (unadjusted HR = 4.08;95% CI = 0.86–19.4;P = 0.08), which was not observed in the subgroup of lymph node positive patients (Table 6). In particular, lymph node negative patients with a germline *BRCA1* variant had a even worse 3-year DFS rate of 77.8%±13.9% than non-carriers (log-rank *P* = 0.02; Fig 2D). Germline *BRCA1* variant was significantly associated with a poor DFS in the lymph node negative subgroup (HR = 5.4;95% CI = 1.12–26.00;*P* = 0.04; Table 6).

**Table 6:** Impact of *BRCA1/2* P/LP variants on the DFS of breast cancer patients from subgroups of N0 and N1~3.

P/LP variants	#Cases (events)	HR (95% CI) ^a^	*P* ^a^	Adjusted HR (95% CI) ^b^	*P* ^b^
**N0**					
**Germline and somatic combined variants**					
**Non-carrier**	165 (9)	1		1	
***BRCA1***	11 (2)	4.08 (0.86–19.4)	0.08	1.7 (0.28–10.32)	0.56
***BRCA2***	12 (0)	0	0.98	0	0.99
**Germline variants**					
**Non-carrier**	165 (9)	1		1	
***BRCA1***	9 (2)	**5.4 (1.12–26.0)**	**0.04**	3.62 (0.47–27.63)	0.21
***BRCA2***	10 (0)	0	0.99	0	0.99
**Somatic variants**					
**Non-carrier**	165 (9)	1		1	
***BRCA1/2***	4 (0)	0	0.70	0	0.99
**N1~N3**					
**Germline and somatic combined variants**					
**Non-carrier**	215 (26)	1		1	
***BRCA1***	8 (1)	1.01 (0.14–7.47)	0.99	0.7 (0.08–6.02)	0.74
***BRCA2***	15 (0)	0	0.98	0	0.98

^a^ Univariate Cox proportional hazards regression models

^b^ Multivariate Cox proportional hazards regression models. HR was adjusted by age at diagnosis, molecular subtype (Luminal A, Luminal B, TNBC, HER2+ and ER-PR+), tumor grade (grade I/II and III) and administration of standard therapy.

#### Overall survival of stage 0~III patients

The stage 0~III patients with *BRCA1/2* P/LP variants shared a similar 3-year OS rate with non-carriers (100% vs. 94.9%±1.4%; log-rank *P* = 0.11). We didn’t find significant association between *BRCA1* or *BRCA2* carriers with overall survival, regardless of their germline or somatic status (Table 4).

## Discussion

This study provided new insights on the complexity of the *BRCA1/2* variants in unselected Chinese breast cancer patients. Germline *BRCA1/2* P/LP variants were detected in 7.9% of the 507 patients. This was higher than the germline *BRCA1/2* mutation rates (2.2%) previously reported in a cohort of 645 unselected Chinese breast cancer women[4], and than that (3.0%) in 471 unselected Korea breast cancer patients using less sensitive methods[23]. In addition, the germline *BRCA2* P/LP variants accounted for 18.8% (3/16) of the FBC patients in our study, and no *BRCA1* variant were detected in FBC subgroup. While in a study with a larger sample size of 99 Chinese patients with hereditary breast cancer, 7.1% in *BRCA1* and 11.1% in *BRCA2* were found by NGS [5].

Using both tumor and paired blood/normal tissue allowed us to identify both somatic and germline mutations. We observed that somatic pathogenic variant accounted for a minor proportion of 1.8% in this cohort (*BRCA1*, 0.6%; *BRCA2*, 1.2%). The percentage was slightly lower than the previous report by The Cancer Genome Atlas (TCGA) using NGS (*BRCA1*, 1.4%; *BRCA2*, 2.0%)[24]. The majority of the races included in TCGA was Caucasian, versus Asian for our study. The genomic diversity may partly explain the various rates of somatic *BRCA* mutation among different races.

Among the 70 TNBC patients, there were 15 (21.4%) *BRCA1/2* P/LP variants carriers (11 with germline *BRCA1*, 1 with somatic *BRCA1*, and 3 with germline *BRCA2*). Our results are in agreement with other studies of unselected TNBC, which demonstrated that germline *BRCA1/2* mutations are enriched in TNBC patients(11.2%-18.2%)[14, 25]. Notably, the next subtype with a high rate of *BRCA1/2* P/LP variants is Luminal B (9.7%, 21 germline and 7 somatic). This is potentially important as Luminal B may not be considered as a high risk group for *BRCA* mutation screening in current clinical practice. Interestingly, the *BRCA1* variants were predominantly found in the TNBC patients, and the *BRCA2* variants were mainly associated with the Luminal B subtype, respectively. We also observed that the germline *BRCA1* P/LP variants have a stronger association with aggressive phenotypes, including triple-negative, tumor grade III, and advanced tumor stage M1. In the contrary, these phenotypes were not observed in the germline *BRCA2* carriers. Our findings are consistent with the previous reports from Asia [26–27] and United States [28] studies.

In patients with stage 0~II disease, we observed a worse DFS in the *BRCA1* (germline or somatic) carriers, as compared to the *BRCA2* carriers or non-carriers. We also found a similar trend in the patients without lymph node involvement. Furthermore, the presence of germline *BRCA1* P/LP variants was significantly associated with an increased risk of relapse in those two subgroups. These findings indicated that the disease progression pattern is diverse among the genetically different breast cancer patients, including those were diagnosed at an early stage. However, we didn’t find association between *BRCA* status and OS, which may need a long-term follow-up.

The impact of germline *BRCA1/2* mutations on patient survival varies in different clinical settings. Moller et al [29] screened the whole coding sequence of *BRCA1/2* in 422 FBC patients, and reported a worse 5-year OS in *BRCA1* carriers, as compared to *BRCA2* carriers or mutation-negative ones. And this prognosis trend maintained when *BRCA1* carriers were diagnosed at an apparently early stage (ex. no lymph involvement or DCIS), which was similar to our observation in DFS. The inferior clinical outcome in *BRCA1* carriers in comparison to *BRCA2* might be explained by aggressive phenotypes [30]. Rennert et al [31] detected three founder *BRCA1/2* mutations in 1317 Israeli women with breast cancer. Consistent with our findings, they found 10-year breast cancer-specific rates of death were similar for carriers of a *BRCA* founder mutation and non-carriers.

On the other hand, in the neoadjuvant chemotherapy setting, TNBC patients with germline *BRCA1* mutation showed a better pathologic complete response to anthracycline with or without taxane regimens [32]. There were also other studies reported that triple-negative breast cancer (TNBC) patients with germline or somatic *BRCA1/2* mutations had a significantly lower risk of relapse[14]. The Cancer Genome Atlas project and University of Washington Medical Center revealed that 6.3%-6.8% of patients with ovarian cancer carried somatic *BRCA1/2* mutation [33–34]. In ovarian cancer patients, somatic mutations together with germline mutations in *BRCA1/2* genes were associated with favorable survival [33, 35]. As showed by preclinical studies, tumor cells with defective homologous recombination as a result of *BRCA* mutation might be more sensitivity to agents that cause DNA strand breaks through intercalation with base pairs (e.g., anthracyclines) or through DNA adduct formation (e.g. platinum) [36–38]. This could partly explain different *BRCA*-associated prognosis observed in the general population and specific clinical settings.

There are some limitations in this study. The sample size herein (n = 507) was relatively small when considering the large diversity of documented variants (ex., over 1500 recorded in BIC database), scattered distribution of these variants in the whole coding regions, and the low frequency of *BRCA1/2* P/LP variants (9.9%). In addition to the variants, to explore methylation and protein expression of *BRCA1/2* genes would further provide us with understanding of the association between *BRCA*ness and patient survival.

In summary, germline or somatic *BRCA1/2* P/LP variants were detected in ~10% of the unselected breast cancer patients from a single hospital in West China. Not only TNBC but also Luminal B subtypes had high frequencies of *BRCA1/2* variants. Germline *BRCA1* carriers tended to have more aggressive tumor phenotypes, including triple-negative, higher tumor grade and advanced disease stage, which was not observed in germline *BRCA2* carriers. On the other hand, patients with somatic *BRCA1/2* variants were likely to be HER2 negative. The presence of a germline or somatic *BRCA1* P/LP variant, especially the germline one, was associated with worse DFS in the patients diagnosed at early stage (Stage 0~II, or N0). These findings suggest that *BRCA1* status is associated with breast cancer phenotype and disease progression, and could be potential prognostic biomarkers especially for early stage disease. It also provided clinical evidence for screening *BRCA1*/*2* status in not only TNBC or FBC, but also Luminal B patients, which are considered as the high risk subgroups.

## Supporting Information

S1 Method Targeted DNA sequencing and bioinformatics analysis. (DOCX)

S1 Table Clinical and pathological characteristics of patients. (DOCX)

S2 Table *BRCA1* and *BRCA2* variants identified by next-generaation sequencing. (DOCX)
