# Supplementary material for: Prevalence and Prognostic Role of BRCA1/2 Variants in Unselected Chinese Breast Cancer Patients
Source: PLoS One. 2016 Jun 3;11(6):e0156789. doi: 10.1371/journal.pone.0156789 (PMC4892623; doi:10.1371/journal.pone.0156789)
Supplement: S1 Method — (DOCX) [file pone.0156789.s001.docx]

**S1 Method. Targeted DNA sequencing and bioinformatics analysis**

DNA was extracted from tumor tissues using Gentra Puregene kit (QIAGEN, Germany). The coding exons and the splice boundaries (-20 /+10 bp) of *BRCA1* and *BRCA2* genes were enriched using *BRCA* MASTR assay v2.1 (Multiplicom, Belgium) following the manufacturer’s instructions. The purified and pooled libraries were sequenced using the MiSeq Reagents v2.0 on a MiSeq system (Illumina, USA). In each run, a no-template control was included as negative control. ArrayStudio v7.2 (OmicSoft, USA) was used for mapping, substitution mutation calling, and indel calling. Variants were filtered by dbSNP138 to remove common SNPs. For large rearrangement screening, customized algorithm was developed for exonic copy number calling. To validate the testing workflow in this study, we used 9 cell lines with known *BRCA1/2* mutations (American Type Culture Collection, Manassas, VA, USA) as positive controls, including HCC1395, HCC1937, MDA-MB-436 cell lines with *BRCA1* mutations, and HCC1569, BT474, CAL51, LS180, HCT116, HCT15 cell lines with *BRCA2* mutations. We also used three commercially available normal DNA samples (Promega, Madison, WI, USA) as negative controls. For patient sample testing, all small variant candidates were successfully validated by Sanger sequencing (ABI 3730XL DNA analyzer, Life Technologies, USA) or the MassARRAY system (Sequenom, USA). Large deletion candidates were confirmed by long-range PCR. Germline or somatic status of all variants was confirmed by testing the paired blood or normal tissue sample using Sanger sequencing. Confirmed variants were then annotated using Alamut software (Interactive biosoftware, France) integrated with multiple databases, including BIC (http://research.nhgri.nih.gov/bic/), UMD (http://www.umd.be/), NCBI ClinVar database (http://www.ncbi.nlm.nih.gov/clinvar/) or LOVD-IARC (http://brca.iarc.fr/LOVD/home.php). Testing and bioinformatics analysis were conducted blinded to clinical information of patients.
