# Supplementary material for: Prevalence and Prognostic Role of BRCA1/2 Variants in Unselected Chinese Breast Cancer Patients
Source: PLoS One. 2016 Jun 3;11(6):e0156789. doi: 10.1371/journal.pone.0156789 (PMC4892623; doi:10.1371/journal.pone.0156789)
Supplement: S1 Table — (DOCX) [file pone.0156789.s002.docx]

**S1 Table. Clinical and pathological characteristics of patients.**

| **Characteristics** |  | **Patient No.** | | **Percentage** |
| --- | --- | --- | --- | --- |
| **Sex** | **Female** | 506 | 99.8% | |
|  | **Male** | 1 | 0.2% | |
| **Ethnic group** | **Han** | 502 | 99.0% | |
|  | **Minority** | 5 | 1.0% | |
| **Age at diagnosis (y)** | **Median, (range)** | 48 (27-84) | | |
|  | **Age＜40** | 64 | 12.6% | |
|  | **40≤age＜60** | 361 | 71.2% | |
|  | **Age≥60** | 82 | 16.2% | |
| **Menopause at diagnosis** | **Postmenopause** | 221 | 43.6% | |
|  | **Premenopause** | 283 | 55.8% | |
|  | **Unknown** | 3 | 0.6% | |
| **Family history of breast cancer** | **Yes** | 16 | 3.2% | |
|  | **No** | 491 | 96.8% | |
| **Family history of ovary cancer** | **Yes** | 0 | 0.0% | |
|  | **No** | 507 | 100.0% | |
| **Bilateral breast cancer** | **Yes** | 5 | 1.0% | |
|  | **No** | 502 | 99.0% | |
| **Histology** | **DCIS** | 5 | 1.0% | |
|  | **Invasive ductal carcinoma** | 453 | 89.3% | |
|  | **Other Invasive carcinomas** | 49 | 9.7% | |
| **WHO grade** | **Ⅰ** | 19 | 3.7% | |
|  | **Ⅱ** | 145 | 28.6% | |
|  | **Ⅲ** | 309 | 60.9% | |
|  | **Unknown** | 34 | 6.7% | |
| **T** | **Tis** | 4 | 0.8% | |
|  | **T1** | 155 | 30.6% | |
|  | **T2** | 305 | 60.2% | |
|  | **T3** | 22 | 4.3% | |
|  | **T4** | 14 | 2.8% | |
|  | **Tx** | 7 | 1.4% | |
| **N** | **N0** | 227 | 44.8% | |
|  | **N1** | 170 | 33.5% | |
|  | **N2** | 61 | 12.0% | |
|  | **N3** | 46 | 9.1% | |
|  | **Nx** | 3 | 0.6% | |
| **M** | **M0** | 501 | 98.8% | |
|  | **M1** | 5 | 1.0% | |
|  | **Mx** | 1 | 0.2% | |

**Table S1. Clinical and pathological characteristics of patients (continued).**

| **Characteristics** |  | **Patient No.** | | **Percentage** |
| --- | --- | --- | --- | --- |
| **Clinical Stage** | **0** | 4 | 0.8% | |
|  | **Ⅰ** | 76 | 15.0% | |
|  | **Ⅱ** | 299 | 59.0% | |
|  | **Ⅲ** | 119 | 23.5% | |
|  | **Ⅳ** | 5 | 1.0% | |
|  | **Unknown** | 4 | 0.8% | |
| **Molecular subtype** | **Luminal A** | 60 | 11.8% | |
|  | **Luminal B** | 290 | 57.2% | |
|  | **HER2+** | 67 | 13.2% | |
|  | **TN** | 70 | 13.8% | |
|  | **ER-PR+** | 20 | 3.9% | |
| **ER** | **Positive（＞1%）** | 359 | 70.8% | |
|  | **Negative** | 148 | 29.2% | |
| **PR** | **Positive（＞1%）** | 344 | 67.9% | |
|  | **Negative** | 163 | 32.1% | |
| **Ki67** | **＜14%** | 82 | 16.2% | |
|  | **≥14%** | 422 | 83.2% | |
|  | **Unknown** | 3 | 0.6% | |
| **HER2** | **Positive** | 154 | 30.4% | |
|  | **Negative** | 353 | 69.6% | |
| **Chemotherapy** | **Yes** | 491 | 96.8% | |
|  | **No** | 16 | 3.2% | |
| **Endocrine therapy for HR+ BC patients** | **Yes** | 341 | 90.7% | |
|  | **No** | 35 | 9.3% | |
| **Anti-HER2 therapy for HER2+ BC patients** | **Yes** | 35 | 22.7% | |
|  | **No** | 119 | 77.3% | |
| **Standard therapy** | **Yes** | 485 | 95.7% | |
|  | **No** | 22 | 4.3% | |
