# Supplementary material for: Prevalence and Prognostic Role of BRCA1/2 Variants in Unselected Chinese Breast Cancer Patients
Source: PLoS One. 2016 Jun 3;11(6):e0156789. doi: 10.1371/journal.pone.0156789 (PMC4892623; doi:10.1371/journal.pone.0156789)
Supplement: S2 Table — (DOCX) [file pone.0156789.s003.docx]

**S2 Table. *BRCA1* and *BRCA2* variants identified by next-generation sequencing. ^a^**

| **Gene** | **Exon** | **Nucleotide change** | **Amino Acid change** | **Category ^b^** | **Novelty ^c^** | **Frequency ^d^** | **Variant Type** | **Somatic/Germline ^e^** |
| --- | --- | --- | --- | --- | --- | --- | --- | --- |
| ***BRCA1*** | 2 | c.66dup | E23INS | P | no | 1 | Frameshift insertion/deletion | Germline |
| ***BRCA1*** | 3 | c.81_134del | C27* | P | no | 1 | Exonic deletion | Germline |
| ***BRCA1*** | 8 | c.519del | Q174DEL | P | yes | 1 | Frameshift insertion/deletion | Germline |
| ***BRCA1*** | 11 | c.981_982del | C328DEL | P | no | 1 | Frameshift insertion/deletion | Germline |
| ***BRCA1*** | 11 | c.3819_3823del | V1274DEL | P | yes | 1 | Frameshift insertion/deletion | Germline |
| ***BRCA1*** | 11 | c.1299dup | S434INS | P | yes | 1 | Frameshift insertion/deletion | Germline |
| ***BRCA1*** | 11 | c.2059C>T | Q687* | P | no | 1 | Nonsense mutation | Germline |
| ***BRCA1*** | 11 | c.2572C>T | Q858* | P | yes | 1 | Nonsense mutation | Germline |
| ***BRCA1*** | 11 | c.2556_2557insTTCACTTTTC | L852INS | P | yes | 1 | Frameshift insertion/deletion | Germline |
| ***BRCA1*** | 11 | c.2570T>A | L857* | P | yes | 1 | Nonsense mutation | Germline |
| ***BRCA1*** | 11 | c.4069_4070insTTGA | Q1356DEL | P | yes | 1 | Frameshift insertion/deletion | Germline |
| ***BRCA1*** | 16 | c.4801A>T | K1601* | P | no | 2 | Nonsense mutation | Germline |
| ***BRCA1*** | 16 | c.4755del | E1586DEL | P | yes | 1 | Frameshift insertion/deletion | Somatic |
| ***BRCA1*** | 16 | c.4712del | F1571DEL | P | yes | 1 | Frameshift insertion/deletion | Germline |
| ***BRCA1*** | 17 | c.5030_5033del | T1677DEL | P | no | 1 | Frameshift insertion/deletion | Germline |
| ***BRCA1*** | 24 | c.5470_5477del | I1824DEL | P | no | 1 | Frameshift insertion/deletion | Germline |
| ***BRCA1*** | 24 | c.5503C>T | R1835* | P | no | 1 | Nonsense mutation | Somatic |
| ***BRCA1*** | 16-17 | c.4676_5074del | E1559_T1691del | P | no | 1 | Exonic deletion | Germline |
| ***BRCA1*** | Intron5 | c.213-2A>G | R71Splicing | P | no | 1 | Splice site mutation | Germline |
| ***BRCA1*** | Intron5 | c.213-1G>A | R71Splicing | P | no | 1 | Splice site mutation | Germline |
| ***BRCA1*** | 5 | c.192T>G | C64W | LP | yes | 1 | Missense mutation | Germline |
| ***BRCA1*** | 8 | c.446A>C | E149A | VUS | no | 1 | Missense mutation | Germline |
| ***BRCA1*** | 11 | c.2268G>T | R756S | VUS | no | 1 | Missense mutation | Germline |
| ***BRCA1*** | 11 | c.837T>C | H279H | VUS | yes | 1 | Synonymous mutation | Germline |
| ***BRCA1*** | 11 | c.3448C>T | P1150S | VUS | no | 1 | Missense mutation | Germline |
| ***BRCA1*** | 11 | c.1819A>G | K607E | VUS | yes | 2 | Missense mutation | Germline |
| ***BRCA1*** | 11 | c.3071G>C | S1024T | VUS | yes | 1 | Missense mutation | Somatic |
| ***BRCA1*** | 11 | c.2726A>T | N909I | VUS | no | 2 | Missense mutation | Germline/Somatic |
| ***BRCA1*** | 11 | c.744C>G | T248T | VUS | no | 1 | Synonymous mutation | Germline |
| ***BRCA1*** | 11 | c.1824_1826del | N609DEL | VUS | yes | 1 | In-frame insertion/deletion | Germline |
| ***BRCA1*** | 11 | c.824G>A | G275D | VUS | no | 1 | Missense mutation | Germline |
| ***BRCA1*** | 11 | c.1392C>T | T464T | VUS | no | 1 | Synonymous mutation | Germline |
| ***BRCA1*** | 11 | c.2387C>T | T796I | VUS | no | 1 | Missense mutation | Germline |
| ***BRCA1*** | 11 | c.2286A>T | R762S | VUS | no | 1 | Missense mutation | Germline |
| ***BRCA1*** | 12 | c.4159T>C | S1387P | VUS | yes | 1 | Missense mutation | Germline |
| ***BRCA1*** | 12 | c.4166G>A | S1389N | VUS | no | 2 | Missense mutation | Germline |
| ***BRCA1*** | 13 | c.4189A>C | R1397R | VUS | yes | 1 | Synonymous mutation | Somatic |
| ***BRCA1*** | 16 | c.4787C>T | S1596L | VUS | no | 1 | Missense mutation | Germline |
| ***BRCA1*** | 16 | c.4704C>T | I1568I | VUS | yes | 1 | Synonymous mutation | Somatic |
| ***BRCA1*** | 16 | c.4803A>G | K1601K | VUS | yes | 1 | Synonymous mutation | Germline |
| ***BRCA1*** | 17 | c.5068A>C | K1690Q | VUS | no | 1 | Missense mutation | Germline |
| ***BRCA1*** | 24 | c.5504G>A | R1835Q | VUS | no | 1 | Missense mutation | Germline |
| ***BRCA1*** | 24 | c.5562del | I1855DEL | VUS | yes | 1 | Frameshift insertion/deletion | Germline |
| ***BRCA2*** | 3 | c.274C>T | Q92* | P | no | 1 | Nonsense mutation | Somatic |
| ***BRCA2*** | 3 | c.161del | H52DEL | P | yes | 1 | Frameshift insertion/deletion | Germline |
| ***BRCA2*** | 10 | c.1399A>T | K467* | P | no | 2 | Nonsense mutation | Germline |
| ***BRCA2*** | 10 | c.1190_1197del | S396DEL | P | yes | 1 | Frameshift insertion/deletion | Somatic |
| ***BRCA2*** | 11 | c.2059_2063del | L686DEL | P | yes | 1 | Frameshift insertion/deletion | Germline |
| ***BRCA2*** | 11 | c.6532dup | I2177INS | P | yes | 1 | Frameshift insertion/deletion | Germline |
| ***BRCA2*** | 11 | c.4148_4149del | D1383DEL | P | no | 1 | Frameshift insertion/deletion | Germline |
| ***BRCA2*** | 11 | c.4151del | D1383DEL | P | no | 1 | Frameshift insertion/deletion | Germline |
| ***BRCA2*** | 11 | c.6402_6406del | N2134DEL | P | yes | 1 | Frameshift insertion/deletion | Germline |
| ***BRCA2*** | 11 | c.2175dup | K725INS | P | no | 1 | Frameshift insertion/deletion | Somatic |
| ***BRCA2*** | 11 | c.2806_2809del | D935DEL | P | yes | 1 | Frameshift insertion/deletion | Germline |
| ***BRCA2*** | 11 | c.4041_4042del | V1347DEL | P | yes | 1 | Frameshift insertion/deletion | Somatic |
| ***BRCA2*** | 11 | c.5645C>A | S1882* | P | no | 2 | Nonsense mutation | Germline |
| ***BRCA2*** | 11 | c.3001del | I1000DEL | P | yes | 1 | Frameshift insertion/deletion | Somatic |
| ***BRCA2*** | 11 | c.5206C>T | Q1736* | P | yes | 1 | Nonsense mutation | Germline |
| ***BRCA2*** | 11 | c.6553del | Q2184DEL | P | no | 1 | Frameshift insertion/deletion | Germline |
| ***BRCA2*** | 11 | c.3834_3835del | H1278DEL | P | yes | 1 | Frameshift insertion/deletion | Germline |
| ***BRCA2*** | 13 | c.6952C>T | R2318* | P | no | 1 | Nonsense mutation | Somatic |
| ***BRCA2*** | 18 | c.8322dup | L2774INS | P | no | 1 | Frameshift insertion/deletion | Somatic |
| ***BRCA2*** | 21 | c.8633_8755del | E2878Gfs*5 | P | no | 1 | Exonic deletion | Germline |
| ***BRCA2*** | 23 | c.9011del | G3003DEL | P | yes | 1 | Frameshift insertion/deletion | Germline |
| ***BRCA2*** | 25 | c.9382C>T | R3128* | P | no | 1 | Nonsense mutation | Germline |
| ***BRCA2*** | 25 | c.9401del | S3133DEL | P | no | 1 | Frameshift insertion/deletion | Germline |
| ***BRCA2*** | 25 | c.9318G>A | W3106* | P | yes | 1 | Nonsense mutation | Germline |
| ***BRCA2*** | 27 | c.9753del | K3251DEL | P | yes | 1 | Frameshift insertion/deletion | Germline |
| ***BRCA2*** | Intron2 | c.67+1G>C | D23Splicing | P | yes | 1 | Splice site mutation | Germline |
| ***BRCA2*** | 20 | c.8632G>A | E2878K | LP | no | 2 | Missense mutation | Germline |
| ***BRCA2*** | 3 | c.92G>T | W31L | VUS | yes | 1 | Missense mutation | Germline |
| ***BRCA2*** | 3 | c.171C>T | Y57Y | VUS | no | 1 | Synonymous mutation | Germline |
| ***BRCA2*** | 4 | c.352C>T | R118C | VUS | no | 1 | Missense mutation | Germline |
| ***BRCA2*** | 5 | c.461A>G | Q154R | VUS | no | 1 | Missense mutation | Germline |
| ***BRCA2*** | 5 | c.454A>G | T152A | VUS | yes | 1 | Missense mutation | Germline |
| ***BRCA2*** | 8 | c.644_646del | E213DEL | VUS | no | 1 | In-frame insertion/deletion | Germline |
| ***BRCA2*** | 10 | c.1568A>G | H523R | VUS | yes | 6 | Missense mutation | Germline |
| ***BRCA2*** | 10 | c.1211A>G | N404S | VUS | no | 2 | Missense mutation | Germline |
| ***BRCA2*** | 10 | c.1591A>G | K531E | VUS | yes | 1 | Missense mutation | Germline |
| ***BRCA2*** | 10 | c.1462A>G | I488V | VUS | no | 1 | Missense mutation | Germline |
| ***BRCA2*** | 10 | c.1786G>A | D596N | VUS | yes | 1 | Missense mutation | Somatic |
| ***BRCA2*** | 10 | c.1511C>G | S504C | VUS | yes | 1 | Missense mutation | Germline |
| ***BRCA2*** | 11 | c.5683G>A | E1895K | VUS | no | 1 | Missense mutation | Germline |
| ***BRCA2*** | 11 | c.6148G>A | V2050I | VUS | no | 1 | Missense mutation | Germline |
| ***BRCA2*** | 11 | c.3473A>G | E1158G | VUS | yes | 1 | Missense mutation | Germline |
| ***BRCA2*** | 11 | c.5590G>A | D1864N | VUS | no | 1 | Missense mutation | Germline |
| ***BRCA2*** | 11 | c.5345A>G | Q1782R | VUS | yes | 1 | Missense mutation | Germline |
| ***BRCA2*** | 11 | c.6598T>C | F2200L | VUS | yes | 1 | Missense mutation | Germline |
| ***BRCA2*** | 11 | c.4376A>G | N1459S | VUS | yes | 1 | Missense mutation | Germline |
| ***BRCA2*** | 11 | c.4915G>A | V1639I | VUS | no | 1 | Missense mutation | Germline |
| ***BRCA2*** | 11 | c.3052A>C | K1018Q | VUS | yes | 1 | Missense mutation | Somatic |
| ***BRCA2*** | 11 | c.6256A>G | I2086V | VUS | yes | 1 | Missense mutation | Somatic |
| ***BRCA2*** | 11 | c.3256A>G | I1086V | VUS | no | 1 | Missense mutation | Germline |
| ***BRCA2*** | 11 | c.5487G>T | L1829F | VUS | yes | 1 | Missense mutation | Germline |
| ***BRCA2*** | 11 | c.6235G>T | V2079L | VUS | yes | 1 | Missense mutation | Germline |
| ***BRCA2*** | 11 | c.4906A>G | K1636E | VUS | no | 1 | Missense mutation | Germline |
| ***BRCA2*** | 11 | c.2136G>T | L712L | VUS | yes | 2 | Synonymous mutation | Germline |
| ***BRCA2*** | 11 | c.2186T>C | I729T | VUS | no | 1 | Missense mutation | Germline |
| ***BRCA2*** | 11 | c.3475T>G | C1159G | VUS | yes | 1 | Missense mutation | Germline |
| ***BRCA2*** | 11 | c.2127G>C | L709L | VUS | yes | 1 | Synonymous mutation | Germline |
| ***BRCA2*** | 11 | c.6325G>A | V2109I | VUS | no | 3 | Missense mutation | Germline |
| ***BRCA2*** | 11 | c.4578A>G | T1526T | VUS | no | 2 | Synonymous mutation | Germline |
| ***BRCA2*** | 11 | c.4548C>G | I1516M | VUS | no | 1 | Missense mutation | Germline |
| ***BRCA2*** | 14 | c.7188G>A | L2396L | VUS | yes | 3 | Synonymous mutation | Germline |
| ***BRCA2*** | 15 | c.7522G>A | G2508S | VUS | no | 2 | Missense mutation | Germline/Somatic |
| ***BRCA2*** | 15 | c.7521A>G | P2507P | VUS | no | 1 | Synonymous mutation | Germline |
| ***BRCA2*** | 15 | c.7545A>G | T2515T | VUS | yes | 1 | Synonymous mutation | Germline |
| ***BRCA2*** | 15 | c.7509C>T | V2503V | VUS | yes | 1 | Synonymous mutation | Germline |
| ***BRCA2*** | 16 | c.7672G>C | E2558Q | VUS | yes | 1 | Missense mutation | Somatic |
| ***BRCA2*** | 16 | c.7772A>G | N2591S | VUS | no | 1 | Missense mutation | Somatic |
| ***BRCA2*** | 17 | c.7967T>C | L2656P | VUS | no | 1 | Missense mutation | Germline |
| ***BRCA2*** | 18 | c.8329A>G | K2777E | VUS | yes | 1 | Missense mutation | Germline |
| ***BRCA2*** | 20 | c.8500A>G | T2834A | VUS | yes | 1 | Missense mutation | Germline |
| ***BRCA2*** | 23 | c.9020G>A | R3007K | VUS | yes | 1 | Missense mutation | unknown |
| ***BRCA2*** | 23 | c.9014G>C | R3005T | VUS | yes | 1 | Missense mutation | Somatic |
| ***BRCA2*** | 25 | c.9285C>T | D3095D | VUS | yes | 2 | Synonymous mutation | Germline |
| ***BRCA2*** | 27 | c.10150C>G | R3384G | VUS | yes | 1 | Missense mutation | Germline |

^a^ The variant data has been submitted to NCBI ClinVar database (<http://www.ncbi.nlm.nih.gov/clinvar/>, Submission ID: SUB1362368).

^b^ Variants were annotated using Alamut software (Interactive biosoftware, France) integrated with multiple databases. Germline variants were interpreted according to the American College of Medical Genetics and Genomics (ACMG). Variants that produce premature termination codons which are associated with non-functional or truncated proteins were classified as pathogenic (P). Some missense mutations were considered as likely pathogenic (LP) variants based on available evidence indicating a strong likelihood of their association with disease. Variants with undetermined clinical significance were classified as variants of uncertain significance (VUS). Similarly, inactivating somatic variants were considered as pathogenic variants, while somatic variants with uncertain clinical significance were considered as VUS.

^c^ Novel variants were defined as variants that have not been previously recorded in BIC (http://research.nhgri.nih.gov/bic/), UMD (http://www.umd.be/), NCBI ClinVar database (http://www.ncbi.nlm.nih.gov/clinvar/) or LOVD-IARC (<http://brca.iarc.fr/LOVD/home.php>), nor reported in the literature.

^d^ Frequency of the variant presented in the 507 breast cancer patients.

^e^ Variants detected in both tumor tissue and paired blood/normal tissue were defined as germline variants. Somatic variants appeared only in tumor tissue, and with a wildtype *BRCA1/2* gene in paired blood/normal tissue. Two variants appeared as germline or somatic variants in different individuals. The germline/somatic status of one *BRCA2* VUS was unknown due to failure of blood detection.
